# Surgical management of focal chondral defects of the knee: a Bayesian network meta-analysis

**DOI:** 10.1186/s13018-021-02684-z

**Published:** 2021-09-01

**Authors:** Filippo Migliorini, Jörg Eschweiler, Hanno Schenker, Alice Baroncini, Markus Tingart, Nicola Maffulli

**Affiliations:** 1 Department of Orthopaedic, Trauma, and Reconstructive Surgery, RWTH University Hospital, Pauwelsstraße 30, 52074 Aachen, Germany; 2 Department of Medicine, Surgery and Dentistry, University of Salerno, Via S. Allende, 84081 Baronissi, SA Italy; 3 Centre for Sports and Exercise Medicine, Mile End Hospital, Barts and the London School of Medicine and Dentistry, Queen Mary University of London, 275 Bancroft Road, London, E1 4DG England; 4 School of Pharmacy and Bioengineering, Keele University Faculty of Medicine, Thornburrow Drive, Stoke on Trent, England

**Keywords:** Knee, Chondral defects, Autologous chondrocyte implantation, Osteochondral autograft transplantation, Autologous matrix-induced chondrogenesis

## Abstract

**Background:**

Focal chondral defects of the knee are common. Several surgical techniques have been proposed for the management of chondral defects: microfractures (MFX), osteochondral autograft transplantation (OAT), autologous matrix-induced chondrogenesis (AMIC) and autologous chondrocyte implantation (ACI)—first generation (pACI), second generation (cACI) and third generation (mACI). A Bayesian network meta-analysis was conducted to compare these surgical strategies for chondral defects in knee at midterm follow-up.

**Methods:**

This Bayesian network meta-analysis was conducted according to the PRISMA extension statement for reporting of systematic reviews incorporating network meta-analyses of health care interventions. PubMed, Google Scholar, Embase and Scopus databases were accessed in July 2021. All the prospective comparative clinical trials investigating two or more surgical interventions for chondral defects of the knee were accessed. The network meta-analyses were performed through a Bayesian hierarchical random-effects model analysis. The log odds ratio (LOR) effect measures were used for dichotomic variables, while the standardized mean difference (SMD) for the continuous variables.

**Results:**

Data from 2220 procedures (36 articles) were retrieved. The median follow-up was 36 (24 to 60) months. The ANOVA test found good baseline comparability between symptoms duration, age, sex and body mass index. AMIC resulted in higher Lysholm score (SMD 3.97) and Tegner score (SMD 2.10). AMIC demonstrated the lowest rate of failures (LOR −0.22) and the lowest rate of revisions (LOR 0.89). As expected, MFX reported the lower rate of hypertrophy (LOR −0.17) followed by AMIC (LOR 0.21). No statistically significant inconsistency was found in the comparisons.

**Conclusion:**

AMIC procedure for focal chondral defects of the knee performed better overall at approximately 3 years’ follow-up.

## Introduction

Focal chondral defects of the knee are common [1]. Avascularity and hypocellularity, along with minimal metabolic activity of cartilage, lead to a limited self-repair capability [2–4]. Chondral defects represent one of the major challenges for orthopaedic surgeons [5]. If left untreated, they negatively impact patient quality of life, reducing their sporting activities and resulting in premature osteoarthritis [6–8]. Knee chondral defects are 20% more common in athletes [9], increasing up to 50% in those who underwent ACL reconstructive surgery [10, 11]. Symptomatic knee chondral defects often require surgery. Microfractures (MFX) represent the traditional approach to these lesions [12]. During osteochondral autograft transplantation (OAT), single or multiple autologous osteochondral grafts are harvested from a donor site and transplanted into the chondral defect [13]. Another surgical technique, namely autologous chondrocyte implantation (ACI), has been in use since 1994 [14]. At ACI, a sample of hyaline cartilage is harvested from a non-weightbearing zone of the distal femur and the chondrocytes are expanded in vitro. In the first generation (periosteal ACI or pACI), expanded chondrocytes are injected into the defect beneath an autologous periosteal membrane [15]. In the second generation (collagenic ACI or cACI), the periosteal membrane is replaced by a collagenic membrane [16]. In the third generation (matrix-induced ACI or mACI), harvested chondrocytes are directly cultivated over a membrane that will then be used to cover the defect [17]. Recently, autologous matrix-induced chondrogenesis (AMIC) has been proposed to manage chondral defect [18, 19]. In AMIC, following MFX of the chondral defect, a membrane is used to cover the lesion in a single step surgery [8, 20]. AMIC exploits the regenerative potential of bone-marrow derived cells. Given the complexity of these injuries, and the number of surgical techniques for knee chondral defects, a Bayesian network meta-analysis was conducted to compare these strategies for the surgical management of focal chondral defects of the knee at midterm follow-up. The purpose of the present study compared efficacy of these strategies in terms of clinical scores and complications.

## Methods

### Search strategy

This Bayesian network meta-analysis was conducted according to the PRISMA extension statement for reporting of systematic reviews incorporating network meta-analyses of health care interventions [21]. The PICOT framework was preliminary pointed out:

- P (Problem): knee chondral defect
- I (Intervention): surgical management
- C (Comparison): pACI, cACI, mACI, AMIC, OAT, MFX
- O (Outcomes): clinical scores and complications
- T (Timing): ≥ 12 months follow-up

### Data source and extraction

Two authors (**;**) independently conducted the literature search. PubMed, Google Scholar, Embase and Scopus databases were accessed in July 2021. The following keywords were used in the database search bar using the Boolean operators AND/OR: *chondral, cartilage, articular, damage, defect, injury, chondropathy, knee, pain, periosteum, membrane, matrix-induced, autologous, chondrocyte, autograft, transplantation, implantation, mACI, pACI, cACI, AMIC, OAT, cylinder, osteochondral, transplantation, autologous matrix-induced chondrogenesis, microfractures, mosaicplasty, management, surgery, outcomes, revision, failures, hypertrophy.* No time constrains were set for the search. The same authors screened separately the resulting articles for inclusion. The full-text of the articles of interest was accessed. A cross reference of the bibliography of the full-text articles was conducted. Disagreements were solved by a third author (**).

### Eligibility criteria

All the clinical trials that compare two or more surgical interventions for knee chondral defects were accessed. Given the authors’ language abilities, articles in English, German, Italian, French and Spanish were eligible. Only prospective studies levels I to II of evidence, according to Oxford Centre of Evidence-Based Medicine [22], were considered. Only studies focusing on AMIC, OAT, MFX and ACI were considered in the present investigation. Only studies that clearly stated the surgical procedures were included. Studies involving patients with end-stage joint osteoarthritis were not eligible, nor were those involving patients with kissing lesions. Only studies reporting data from procedures in knee with a minimum 12 months follow-up were eligible. Animals and computational studies were not considered. Studies augmenting the intervention with less committed cells (e.g. mesenchymal stem cells) were not considered. Missing quantitative data under the outcomes of interest warranted the exclusion from this study.

### Outcomes of interest

Two authors (**;**) separately performed data extraction. Study generalities (author, year, journal, type of study) and patients’ baseline demographic information were extracted (number of samples and related mean BMI and age, duration of the symptoms, duration of the follow-up, percentage of female). For every study, data concerning the International Knee Documentation Committee (IKDC) [23], Tegner Activity Scale [24] and Lysholm Knee Scoring Scale [25] at last follow-up was collected. Data regarding complications were also collected: hypertrophy, rate of failures and revisions. Failure was defined as pain and/or catching symptoms recurrence, partial or complete displaced delamination at MRI or arthroscopy [26–28].

### Methodology quality assessment

The methodological quality assessment was performed by two authors (**;**). The risk of bias graph tool of the Review Manager Software (The Nordic Cochrane Collaboration, Copenhagen) was used. The following risks of bias were evaluated: selection, detection, reporting, attrition and other source of bias.

### Statistical analysis

The statistical analysis was performed by the main author (**). The STATA Software/MP (StataCorporation, College Station, TX, USA) was used for the statistical analyses. To assess demographic baseline, the Shapiro-Wilk test has been performed to investigate data distribution. For parametric data, mean and standard deviation were evaluated. The baseline comparability was assessed using analysis of variance (ANOVA), with *P* values > 0.1 considered satisfactory. For non-parametric data, median and interquartile were evaluated. The baseline comparability was assessed by the Kruskal-Wallis test, with *P* values > 0.1 considered satisfactory. The network meta-analyses were performed through the STATA routine for Bayesian hierarchical random-effects model analysis. The inverse variance method was used for all the comparisons. The log odds ratio (LOR) effect measures were used for dichotomic variables, while the standardized mean difference (SMD) for the continuous variables. The overall inconsistency was evaluated through the equation for global linearity via the Wald test. If *P* value > 0.1, the null hypothesis could not be rejected, and the consistency assumption is accepted at the overall level of each treatment. All the variables were compared in the network analyses against a fictitious group control: no event for binary comparisons and maximal value of score for continuous endpoints. Both confidence (CI) and percentile (PrI) intervals were set at 95%. Edge plots, interval plots and funnel plots were obtained and evaluated.

## Results

### Search result

**Fig. 1:**
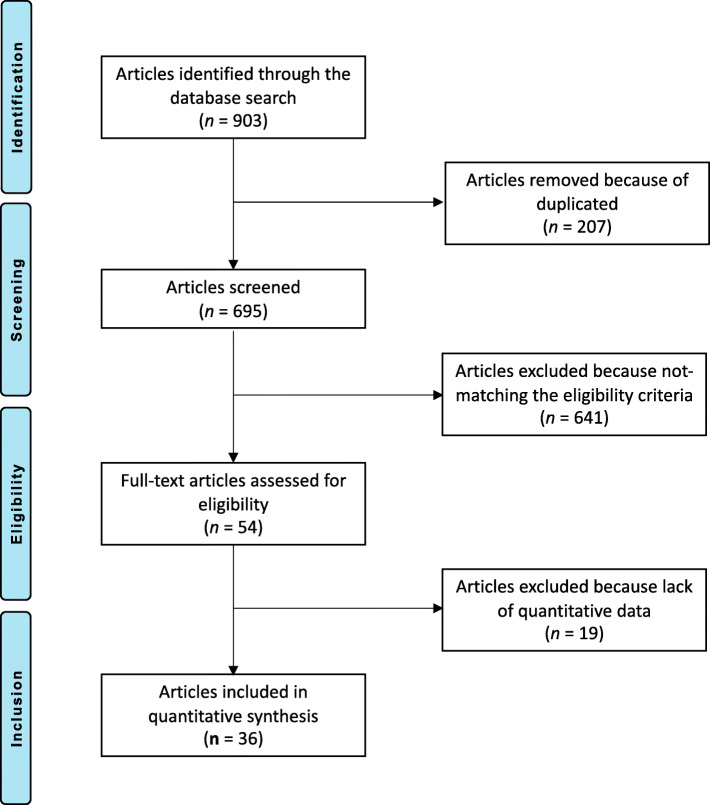
Flow chart of the literature search

The literature search resulted in 903 articles. Of them, 207 were duplicates. A further 641 articles did not match the inclusion criteria: poor level of evidence or not comparative study (*N* = 407), not focused on knee (*N* = 197), reported short follow-up (*N* = 9), combined with stem cells (*N* = 11) and language limitations (*N* = 2). A further 15 articles were excluded since they did not clearly specify the surgical procedure or the eligibility criteria. A further 19 studies were not considered because they did not report quantitative data under the outcomes of interest. This left 36 comparative studies: 22 RCTs and 14 non-RCTs. The literature search results are shown in Fig. 1.

### Methodological quality assessment

**Fig. 2:**
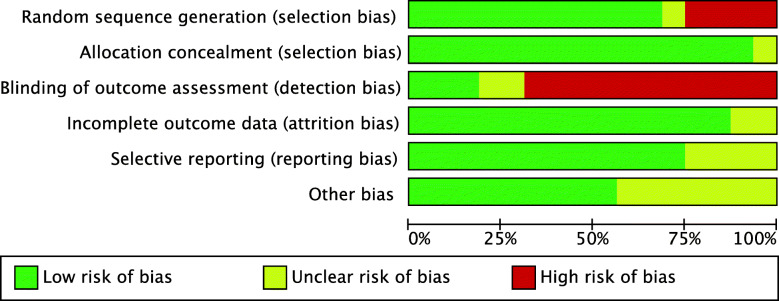
Methodological quality assessment: Cochrane risk of bias graph

Given the predominance of RCTs (22 of 36 studies), the risk to selection bias was low. The risk of selection bias of the allocation concealment was very low. Given the overall lack of blinding, the risk of detection bias was moderate to high. The risk of attrition and reporting bias were low, as were the risks of other biases. Concluding, the overall review authors’ judgements about each risk of bias item scored low, attesting to this study a good methodological assessment. The risk of bias graph is shown in Fig. 2.

### Patient demographics

Data from 2220 procedures were retrieved. The mean duration of symptoms before the index surgery was 44 (25 to 86.5) months. Thirty-six percent (799 of 2210) were women. The median age of the patients was 33.9 (30 to 37) years, while the median BMI was 25.3 (25 to 26) kg/m^2^. The mean defect size was 3.7 ± 1.2 cm^2^. The median follow-up was 36 (24 to 60) months. The ANOVA test found good between studies baseline comparability in terms of mean duration of symptoms, age, BMI, gender, defect size and preoperative VAS, Tegner, Lysholm and IKDC (P > 0.0.5). Generalities of the study are shown in Table 1, while the within studies baseline is shown in greater detail in Table 2.

**Table 1:** Generalities of the included studies

Author, year	Journal	Study design	Follow-up (months)	Treatment	Procedures (n)	Female (%)	Mean age	Mean BMI
Anders et al., 2013 [33]	*Open Orthop J*	Randomized	24	AMIC	8	12	35.0	27.4
				AMIC	13	23	39.0	27.7
				MFX	6	33	41.0	25.2
Bartlett et al., 2005 [16]	*J Bone Joint Surg*	Randomized	12	cACI	44	41	33.7	
				mACI	47		33.4	
Basad et al., 2010 [62]	*Knee Surg Sports Traumatol Arthrosc*	Randomized	24	mACI	40	38	33.0	25.3
				MFX	20	15	37.5	27.3
Becher et al., 2017 [63]	*J Orthop Surg Res*	Randomized	36	mACI	25	32	33.0	24.9
				mACI	25	16	34.0	25.6
				mACI	25	40	34.0	25.1
Berruto et al., 2017 [64]	*Injury*	Non-Randomized	162	pACI	9	31	31.6	
				cACI	24			
Bode et al., 2013 [65]	*Arch Orthop Trauma Surg*	Non-Randomized	72	cACI	19		40.2	25.2
				cACI	24		38.3	24.1
Brittberg et al., 2018 [66]	*Am J Sports Med*	Randomized	60	mACI	65	38	35.0	
				MFX	63	33	34.0	
Chung et al., 2013 [32]	*Knee Surg Sports Traumatol Arthrosc*	Non-Randomized	24	MFX	12	83	44.3	
				AMIC	24	42	47.4	
Cvetanovich et al., 2016 [67]	*Am J Sports Med*	Non-Randomized	24	cACI	12	22	17.0	22.8
				mACI	11	22	17.0	22.8
				mACI	14	22	17.0	22.8
De Girolamo et al., 2019 [68]	*J Clin Med*	Randomized	100	AMIC	12	39	30.0	
				AMIC	12	40	30.0	
Ebert et al., 2015 [69]	*Am J Sports Med*	Non-Randomized	24	mACI	10	20	39.0	25.8
				mACI	13	7	36.0	25.6
				mACI	9	66	38.0	25.1
				mACI	15	53	37.0	25.3
Ferruzzi et al., 2008 [70]	*J Bone Joint Surg*	Non-Randomized	60	pACI	48	38	32.0	
				mACI	50	28	31.0	
Fossum et al., 2019 [30]	*Orthop J Sports Med*	Randomized	24	AMIC	20	60	38.3	27.9
				cACI	21	33	37.2	25.7
Gooding et al., 2006 [71]	*Knee*	Randomized	24	pACI	33	51	31.0	
				cACI	35			
Gudas et al., 2006 [72]	*Knee Surg Sports Traumatol Arthrosc*	Randomized	37	MFX	28	43	24.3	
				OAT	29	35	24.6	
Gudas et al., 2009 [73]	*J Pediatr Orthop*	Randomized	24	OAT	25	40	15.0	
				MFX	22	40	14.0	
Gudas et al., 2012 [74]	*Am J Sports Med*	Randomized	120	OAT	28	32	25.0	
				MFX	29	41	24.0	
Hoburg et al., 2019 [28]	*Orthop J Sports Med*	Non-Randomized	63	mACI	29	48	16.0	21.3
			48	mACI	42	29	27.0	24.1
Horas et al., 2003 [75]	*J Bone Joint Surg*	Non-Randomized	124	pACI	20	60	31.4	
				OAT	20	25	35.4	
Knutsen et al., 2016 [76]	*J Bone Joint Surg*	Randomized	180	pACI	40			
				MFX	40			
Kon et al., 2009 [42]	*Am J Sports Med*	Non-Randomized	60	mACT	40	17	29.0	
				MFX	40	32	31.0	
Kon et al., 2011 [43]	*Am J Sports Med*	Non-Randomized	61	mACT	22	32	46.0	24.7
			58	mACI	39	35	45.0	25.6
Lim et al., 2012 [77]	*Clin Orthop Rel Res*	Randomized	60	MFX	30	40	33.0	
				OAT	22	45	30.0	
				pACI	18	44	25.0	
Macmull et al., 2012 [78]	*Int Orthop*	Non-Randomized	66	cACI	24	29	16.0	
				mACI	7			
Macmull et al., 2012 [79]	*Am J Sports Med*	Non-Randomized	45	cACI	25	80	35.0	
			35	mACI	23	61	35.0	
Niemeyer et al., 2016 [80]	*Am J Sports Med*	Randomized	12	mACI	25	33	33.0	24.9
				mACI	25	16	34.0	25.6
				mACI	25	40	34.0	25.1
Niemeyer et al., 2019 [27]	*Orthop J Sports Med*	Randomized	24	mACI	52	36	36.0	25.7
				MFX	50	44	37.0	25.8
Saris et al., 2009 [81]	*Am J Sports Med*	Randomized	36	pACI	57	39	33.9	
				MFX	61	33	33.9	
Saris et al., 2014 [82]	*Am J Sports Med*	Randomized	24	mACI	72	37	35.0	26.2
				MFX	72		33.0	26.4
Schneider et al., 2016 [83]	*J Orthop Surg*	Randomized	12	MFX	13	50	47.0	
				MFX	4		37.0	
Skowronski et al., 2013 [55]	*Orthop Traumatol Rehab*	Non-Randomized	60	cACI	21	42	26.0	
				cACI	25	44	26.0	
Van Assche et al., 2010 [84]	*Knee Surg Sports Traumatol Arthrosc*	Randomized	24	pACI	33	33	31.0	24.0
				MFX	34	10	31.0	25.0
Vanlauwe et al., 2011 [85]	*Am J Sports Med*	Randomized	60	MFX	61	20	34.0	
				pACI	51	43	34.0	
Volz et al., 2017 [31]	*Int Orthop*	Randomized	60	AMIC	17	29	34.0	27.4
				AMIC	17	11	39.0	27.6
				MFX	13	23	40.0	25.0
Wolf et al., 2018 [86]	*Cartilage*	Non-Randomized	24	MFX	18	55	38.0	
				MFX	3		50.0	
Zeifang et al., 2010 [87]	*Am J Sports Med*	Randomized	24	mACI	11	45	29.0	
				pACI	10	0	30.0

**Table 2:** Patient demographic at baseline

Treatment	AMIC (***N*** = 103)	cACI (***N*** = 253)	mACI (***N*** = 761)	MFX (***N*** = 619)	OAT (***N*** = 124)	pACI (***N*** = 319)
Follow-up (*months*)	56.0 ± 34.1	59.7 ± 42.0	44.9 ± 18.2	45.7 ± 40.6	73.0 ± 46.6	75.4 ± 58.7
Female (*%*)	29.2 ± 14.9	43.0 ± 20.1	33.8 ± 14.4	37.2 ± 17.4	35.3 ± 7.6	37.7 ± 16.7
Mean age	36.3 ± 6.1	29.0 ± 9.3	32.7 ± 7.3	34.9 ± 8.4	26.0 ± 7.6	31.1 ± 2.6
Mean BMI	27.5 ± 0.2	24.0 ± 1.2	24.8 ± 1.2	25.8 ± 0.9	26.1 ± 1.1	24.0 ± 1.3
defect size (*cm*^*2*^)	3.4 ± 0.9	4.8 ± 0.7	4.2 ± 1.1	2.7 ± 0.9	3.1 ± 0.4	3.9 ± 1.4
Symptoms		83.6 ± 31.0	64.8 ± 30.2	30.6 ± 10.1	23.5	47.4 ± 27.1
VAS (*0–10*)	6.1 ± 0.5	5.9 ± 0.5	6.3 ± 0.4	6.1		4.8
Tegner score	4.5 ± 0.3		3.1 ± 1.6	2.4 ± 0.6	2.7	3.4 ± 1.0
Lysholm score	68.8 ± 5.0	53.6 ± 1.6	61.7 ± 13.7	53.5 ± 2.2	53.2	56.9 ± 6.3
IKDC score	47.0	36.3	37.7 ± 6.9	36.0 ± 6.5		46.2 ± 8.3

### Outcomes of interest

**Fig. 3:**
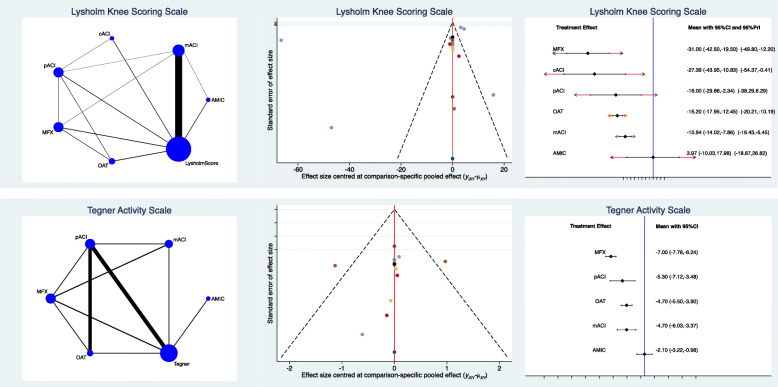
Results of Tegner and IKDC scores. The edge plot (*left*) showed direct and indirect comparisons; greater asymmetries of estimated effects in the funnel plot (*middle*) correlated with higher risk of bias; the interval plot (*right*) ranked the final effects of the network comparisons

AMIC reported higher Lysholm score (SMD 3.97; 95% CI −10.03 to 17.98) and Tegner score (SMD 2.10; 95% CI −3.22 to −0.98). No statistically significant heterogeneity was found concerning these two endpoints (*P* > 0.1). Statistically significant inconsistency was found for the comparison IKDC; therefore, no further considerations can be inferred. Edge, funnel and interval plots of the Lysholm and Tegner scores are shown in Fig. 3.

### Complications

**Fig. 4:**
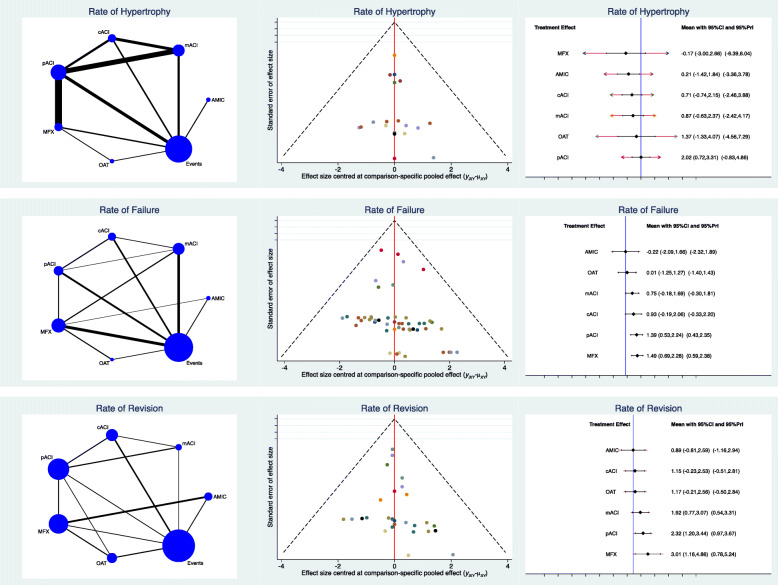
Results of complications. The edge plot (*left*) showed direct and indirect comparisons; greater asymmetries of estimated effects in the funnel plot (*middle*) correlated with higher risk of bias; the interval plot (*right*) ranked the final effects of the network comparisons

AMIC demonstrated the lowest rate of failures (LOR −0.22; 95% CI −2.09 to 1.66) and the lowest rate of revisions (LOR 0.89; 95% CI −0.81 to 2.59). As expected, MFX showed the lowest rate of hypertrophy (LOR −0.17; 95% CI −3.00 to 2.66) followed by AMIC (LOR 0.21; 95% CI −1.42 to 1.84). No statistically significant inconsistency was found concerning these two endpoints (*P* > 0.1). Edge, funnel and interval plots of complications are shown in detail in Fig. 4.

## Discussion

According to the present Bayesian network meta-analysis, AMIC procedure for the management for chondral defects of the knee performed better overall at approximately 3 years’ follow-up. Among the ACI procedures, mACI performed better. Patients undergoing pACI reported the highest rate of graft hypertrophy, while MFX performed worst overall.

To the best of our knowledge, only Riboth et al. in 2016 [29] conducted a Bayesian network meta-analysis on surgical strategies for chondral defect of knee. Their study was based on 15 RCTs, involving 855 procedures. In the present study, the number of procedures was greater, as we identified for analysis 21 RCTs and 14 prospective cohort studies with level of evidence II. Differently to Riboth et al. [29], we also implemented the analyses including the rate of failure, included AMIC procedures and analysed separately the results of the Tegner and Lysholm scores. The current literature lacks head-to-head studies that compared AMIC with other surgical techniques for the management of knee chondral defects. AMIC is a single stage technique that avoids the harvesting of non-weightbearing cartilage, cells culture and expansion, exploiting the potential of autologous bone marrow-derived mesenchymal stem cells (MSCs). The nature of the membrane used for AMIC is the same of mACI. Fossum et al. [30] comparing 20 patients treated with AMIC versus 21 patients with cACI, at 2 years’ follow-up, reported no significant differences between the two techniques in terms of Knee injury and Osteoarthritis Outcome Score (KOOS), Lysholm, VAS and rate of TKA. Previous studies have compared AMIC versus MFX for knee chondral defects. Volz et al. [31] compared AMIC versus MFX at 5 years postoperatively. AMIC was an effective cartilage repair procedure with stable clinical results and significantly greater outcome scores than the MFX group [31]. Similar results were found by Chung et al. [32] and Anders et al. [33] at 2 years’ follow-up.

The present Bayesian network meta-analysis certainly has limitations. The limited number of studies and consequently procedures is an important limitation. Chondrocyte culture and expansion methods for ACI among the included studies are heterogeneous. We included all types of surgical approach (arthroscopy, mini-open, arthrotomy), membrane type (collagenic or hyaluronic) and fixation (glue, fibrin, both, none). The influence of these factors has not been yet fully clarified, and further studies are required. Several comparative trials concerning MSCs augmentation for knee chondral defects have been published [34–38]. While MSCs seem to hold great potential for musculoskeletal systems [39–41], to overcome current limitations to clinical translation is still challenging and a deeper understanding of the biological background to optimize tissue neogenesis is required. Thus, given these limitations, studies concerning MSC augmentation were not considered for inclusion. Two studies [42, 43] performed membrane-assisted autologous chondrocyte transplantation (mACT). In the mACT technique, chondrocytes are cultivated and expanded into a membrane in the same fashion of mACI. The chondrocyte-loaded membrane is then carefully transplanted to fill the defect with custom-made instruments in a full-arthroscopic fashion [44, 45]. We included data from this technique in the mACI group and did not analyse them separately. Given the lack of data, it was not possible to analyse the aetiology of chondral defects as separate data sets. Moreover, almost all the included studies did not analyse primary and revision surgeries as separate events. Similarly, most of studies reported data over multiple locations, without differentiation between patella, trochlear, condylar and tibial defects. Finally, many authors combined these techniques with other surgical intervention, such as osteotomy, tibial tubercle transfer and meniscal procedures, and data were not presented separately. Given these limitations, results from the present study should be interpreted with caution. Current evidence concerning chondral procedures augmented with mesenchymal stem cells (MSCs) is still very limited [34–38, 46–55]. The best delivery protocol is still debated, and several different procedures are described through different methodologies with a variable degree of invasiveness, from arthroscopy to mini arthrotomy, or formal arthrotomy [34–36, 46–50, 52–54]. Most articles investigating chondral procedures augmented with MSCs referred to a small sample size and limited length of the follow-up, and the size and location of the chondral defect and the cell delivery protocol are heterogeneous, precluding statistical analysis [1, 56–61]. Moreover, meniscectomy, synovectomy, anterior cruciate ligament repair and high tibial osteotomy were often performed concomitantly [34–38, 46, 50, 52, 53]. Several MSCs sources, culture, expansion and implantation modalities have been described, but seldom compared to one another. Thus, given these limitations, chondral procedures augmented with MSCs were not included. Future studies should overcome these limitations to give new insights and more reliable results.

## Conclusion

AMIC procedure as management for focal chondral defects of the knee performed better overall at approximately 3 years’ follow-up.

## Data Availability

This study does not contain any third material.

## Abbreviations

MFX: Microfractures
OAT: Osteochondral autograft transplantation
AMIC: Autologous matrix-induced chondrogenesis
ACI: Autologous chondrocyte implantation
pACI: Periosteal autologous chondrocyte implantation
cACI: Collagen-membrane autologous chondrocyte implantation
mACI: Matrix-induced autologous chondrocyte implantation
LOR: Log odds ratio
SMD: Standardized mean difference
